# The impact of treatment facility type on the survival of brain metastases patients regardless of the primary cancer type

**DOI:** 10.1186/s12885-021-08129-4

**Published:** 2021-04-09

**Authors:** Saber Amin, Michael Baine, Jane Meza, Chi Lin

**Affiliations:** 1 Department of Radiation Oncology, University of Nebraska Medical Center, 986861 Nebraska Medical Center, Omaha, NE 68198-6861 USA; 2 Department of Biostatistics, College of Public Health, University of Nebraska Medical Center, Omaha, USA

**Keywords:** Brain metastases, Treatment facility, Overall survival, Radiation therapy, Surgery

## Abstract

**Background:**

Cancer patients with brain metastases (BMs) require multidisciplinary care, and treatment facility may play a role. This study aimed to investigate the impact of receiving treatment at academic centers on the overall survival (OS) of cancer patients with brain metastases (BMs) regardless of the primary cancer site.

**Methods:**

This retrospective analysis of the National Cancer Database (NCDB) included patients diagnosed with non-small cell lung cancer, small-cell lung cancer, other types of lung cancer, breast cancer, melanoma, colorectal cancer, and kidney cancer and had brain metastases at the time of diagnosis. The data were extracted from the de-identified file of the NCDB, a joint program of the Commission on Cancer of the American College of Surgeons and the American Cancer Society. The Cox proportional hazard model adjusted for age at diagnosis, race, sex, place of living, income, education, primary tumor type, year of diagnosis, chemotherapy, radiation therapy (RT), and surgery of the primary cancer site was used to determine treatment facility-associated hazard ratios (HR) for survival. Overall survival was the primary outcome, which was analyzed with multivariable Cox proportional hazards regression modeling.

**Results:**

A total of 93,633 patients were analyzed, among whom 31,579/93,633 (34.09%) were treated at academic centers. Based on the log-rank analysis, patients who received treatment at an academic facility had significantly improved OS (median OS: 6.18, CI: 6.05–6.31 vs. 4.57, CI: 4.50–4.63 months; *p* < 0.001) compared to patients who were treated at non-academic facilities. In the multivariable Cox regression analysis, receiving treatment at an academic facility was associated with significantly improved OS (HR: 0.85, CI: 0.84–0.87; *p* < 0.001) compared to non-academic facility.

**Conclusions:**

In this extensive analysis of the NCDB, receiving treatment at academic centers was associated with significantly improved OS compared to treatment at non-academic centers.

**Supplementary Information:**

The online version contains supplementary material available at 10.1186/s12885-021-08129-4.

## Background

It is estimated that each year more than 170,000 people are newly diagnosed with brain metastases (BMs) in the United States [1]. Brain metastases are ten times more common than the primary intracranial cancer and represent the most common intracranial malignancy in adults [2–5]. The most common primary tumors associated with BMs are lung (40–50%), breast (15–30%), and melanoma (5–20%), followed by colorectal cancer (CRC) (3–8%), and renal cell cancer (2–4%) [6]. Brain metastases are associated with significant morbidity and mortality and carry a poor survival prognosis [7]. The median overall survival (OS) of BMs patients depends on the primary cancer site and ranges between 4 and 16 months [8–10].

The current treatment modalities available for the treatment of BMs are surgery, whole-brain radiation therapy (WBRT), and stereotactic radiosurgery (SRS) [11, 12]. Targeted therapies and immunotherapies were associated with improved OS and intracranial response rate in BMs patients from melanoma, breast, non-small cell lung cancer (NSCLC), and renal cell carcinoma [13–20]. HER2 inhibitor was associated with an objective central nervous system response rate of 74% and a median OS of 10.5 months [95% CI, 7.8–13.2] in BMs patients from breast cancer [16]. Epidermal growth factors receptor inhibitor was associated with 3 months of improved median OS compared to chemotherapy in patients with BMs from NSCLC [12 vs. 9 months] [17]. Pembrolizumab was associated with a response rate of 33% [95% CI, 14–59)] in BMs patients from NSCLC and 22% [95% CI, 7–48)] in BMs from melanoma patients [21]. The combination of nivolumab and ipilimumab was associated with 56% complete or partial intracranial response [22].

Nevertheless, proper management of brain metastasis requires multidisciplinary input about the appropriate integration of surgery, radiation, and systemic therapies. Furthermore, the quality of life of patients and the long-term toxicity and complications of the treatments should also be carefully weighed when deciding on the treatment of BMs patients. These therapeutic challenges require advanced multidisciplinary care and access to a robust health care team. Due to the highly specialized and interdisciplinary treatment approach being needed for BMs, hospital teaching status is to contribute to variation in patient survival outcomes. In addition to institutional variables such as the technical ability, presence of a robust and experienced health care team, and novel treatment modalities offered, certain patients related factors such as race, education, income, insurance can vary between academic and non-academic facilities and may affect the survival outcomes.

Studies of various malignancies have indicated that the choice of treatments and survival outcomes varies by academic vs. non-academic hospitals. A study found that academic centers used stereotactic radiosurgery (SRS) more frequently (22% versus 13%, *p* < .001) compared to community facilities for brain metastasis from NSCLC [23]. A meta-analysis of head and neck cancer patients who received surgical resection of the tumor examined survival outcomes between patients treated at high volume vs. low volume centers and demonstrated better overall survival among patients treated by high-volume hospitals than among patients treated by low-volume hospitals [24]. Treatment at academic hospitals was associated with Improved overall survival with a hazard ratio (HR: 0.89, CI: 0.88–0.91) compared to community center programs in a study of head and neck cancer patients who received surgery. In this study, patients with Medicaid and patients from low-income areas were less likely to receive treatment at academic centers [25]. Few studies of the Glioblastoma patients who underwent surgery reported improved OS for patients treated at academic centers compared to community treatment centers [26–28]. Better OS associated with receiving treatment at academic facilities has also been reported in resectable pancreatic cancer, intrahepatic cholangiocarcinoma, and metastatic NSCLC [29–31].

Due to the complex treatment modalities and expertise needed for the treatments of BMs patients, the impact of academic or research treatment facilities on the OS of BMs patients must be investigated. There have been no studies that have compared the OS of BMs patients regardless of the primary cancer site between academic and non-academic hospitals. The objective of this study is to examine the difference between the OS of BMs patients who receive treatment at academic hospitals and those who receive treatment at non-academic hospitals using the National Cancer Database (NCDB).

## Methods

### Data source and patient cohort

The data for this retrospective analysis were extracted from the National Cancer Database (NCDB), a nationwide oncology outcomes database for more than 1500 Commission-accredited cancer programs in the United States and Puerto Rico, which captures 70% or more of newly diagnosed malignancies in the United States. The NCDB is a multi-centers hospital-based cancer registry that was established in 1989 and now contains approximately 34 million records from hospital cancer registries across the United States. The data are extracted from patient charts by Certified Tumor Registrars, who undergo training specific to cancer registry operations. This study was exempt from the institutional review board as the de-identified data were used, and no consent was required.

### Study population

Patients age 18 or older, who had brain metastases at the time of diagnosis and were diagnosed with the primary cancer of NSCLC, small-cell lung cancer (SCLC), other types of lung cancer, breast cancer, colorectal cancer, melanoma, and kidney cancer between 2010 and 2015, were included in this study. Patients with M0, patients who were missing information about M stage, treatment facility, surgery to the primary site, RT, chemotherapy, and immunotherapy were excluded from the analysis. Per the NCDB definition, an academic facility is defined as an institution that must have more than 500 newly diagnosed cancer cases per year and are associated either with a National Cancer Institute-designated comprehensive cancer center or provide postgraduate medical education in at least four program areas, including internal medicine and general surgery. All other facilities, including the Community Cancer Program, Comprehensive Community Cancer Program, and Integrated Network Cancer Program, were combined and considered as non-academic facilities as none of these institutions require graduate medical education. Comprehensive community centers have > 500 cancer cases diagnosis/year, while community cancer centers have between 100 and 500 new cancer diagnosis cases/year. An integrated network cancer program is a network of multiple facilities that work together to provide integrated care [32, 33]. The surgery variable is surgery to the primary site. Some patients may have received brain surgery, but that information is not provided in the NCDB. The RT is radiation therapy to any site, which could be RT to the brain, RT to other sites, or both. The income is based on the income level of the zip code where the patient lives. The same is true for the education variable. It represents the proportion of people with no high school degree from the zip code where the patient lives. These two variables are estimated by matching the zip code of the patient recorded at the time of diagnosis against files derived from the 2012 American Community Survey data, spanning the years 2008–2012. All study patients had brain metastases at the time of diagnosis of primary cancer.

### Outcomes of interest

The primary outcome of the current study was to determine the OS of the patients, which was measured in months and calculated from the date of diagnosis to the date of death. Those alive or lost to follow up were censored. The odds ratio for patients and tumor-related factors associated with the probability of receiving treatment at academic hospitals were also reported.

### Explanatory variables

The primary explanatory variable was the treatment facility. Other variables were age at diagnosis, sex, race, place of living, income, education, hospital type, comorbidity score, insurance status, primary tumor type, year of diagnosis, and receipt of chemotherapy, surgery, radiation therapy, and immunotherapy.

### Statistical analyses

Descriptive statistics for categorical and continuous variables were reported. The multiple logistic regression analysis was implied to determine the factors associated with receiving treatment at academic facilities. The Odds ratio was reported as the measure of association with the likelihood of being treated at an academic facility. The Kaplan-Meier method was used to generate survival curves and compare OS between groups using the log-rank test. The Cox proportional hazard regression analysis was conducted to estimate hazard ratios and their associated 95% confidence intervals. The Multivariable Cox regression model included all variable variables from the univariate Models as all of them were significant. The backward elimination technique was used to develop the final model, and variables with a *p*-value of < 0.05 remained in the final model. The PH assumptions were tested and verified by log-log plots. There was no violation of PH assumptions. The *p*-value of 0.05 was considered significant. The SAS 9.4 software from SAS Inc. was used for the analysis.

## Results

### Patient and treatment characteristics

**Fig. 1:**
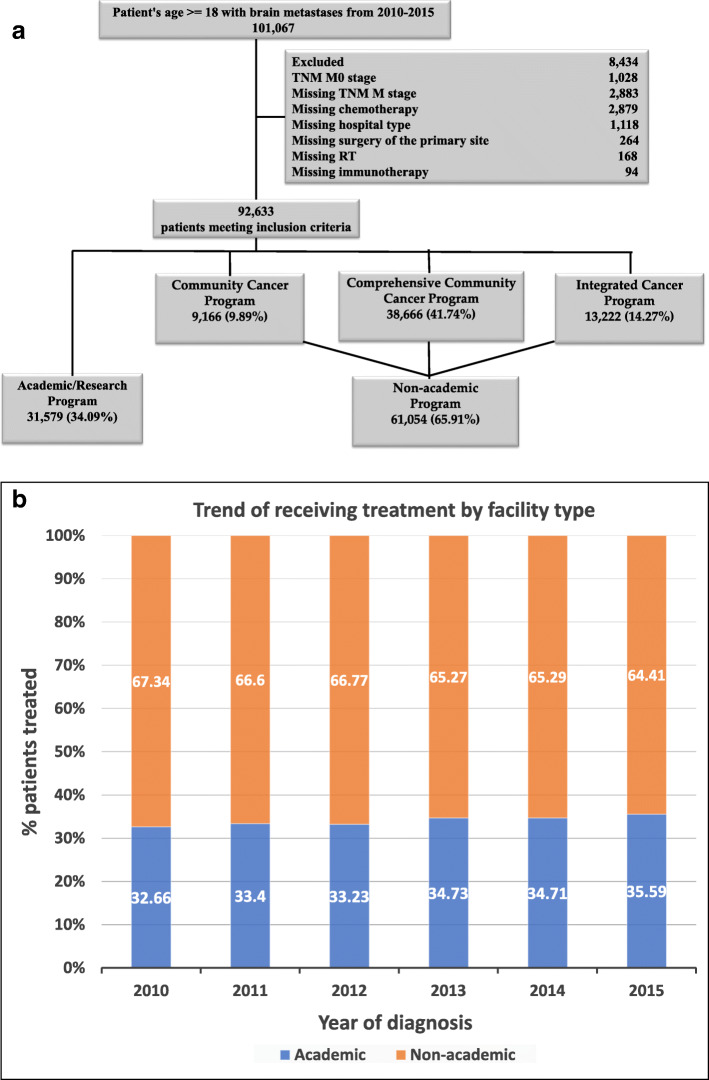
**a** Patient selection diagram. **b** The trend of receiving treatment at academic and non-academic hospitals over time

Data were obtained from NCDB for 101,067 patients with BMs diagnosed between 2010 and 2015. Patients excluded included those who were M0 stage or were missing information related to the M stage, facility type, and treatment variables (8434) (Fig. 1a). The final analysis included 93,633 patients, among whom 31,579 (34.09%) were treated at academic facilities, and 61,054 (65.91%) were treated at non-academic treatment facilities. There were 1317 facilities, among which 226/1317 (17.2%) were academic facilities, and the remaining 1091/1317 (82.8%) were non-academic facilities. On average, each academic facility treated 23.3 cases/year, while each non-academic facility treated 9.3 cases/year. The median age of the entire study population was 65 with a range of (40–90) years. The median age at diagnosis of the patients treated at academic facilities was 64 (40–90), while the median age at diagnosis of the patients treated at non-academic facilities was 66 (40–90). The majority of the patients were White, from high income-level areas, had insurance, did not receive surgery of the primary site, received RT, had a comorbidity score of zero, were diagnosed between 2010 and 2013, and had NSCLC. The trend of receiving treatment at academic and non-academic hospitals over time is illustrated in Fig. 1b.

### Outcomes

Younger age, black race, non-white non-black, living in urban areas, living in areas with a high proportion of people with no high school degree, living in areas with high income-level, having comorbidity score of zero, receiving surgery of the primary tumor, RT, chemotherapy, immunotherapy, and diagnosis in 2014 or after were more likely to be treated at academic facilities compared to non-academic facilities. Patients belonging to areas with income <$35,000 were 16% less likely to receive treatment at an academic facility (OR: 0.839, CI: 0.811–0.868) compared to their counterparts from the areas with income = > $35,000. Patients from rural counties were 27% less likely to receive treatment at academic facilities (OR: 0.726, CI: 0.657–0.803) compared to patients from urban areas. Patients who did not undergo surgery of the primary site tumor were 10% less likely (OR: 0.90, CI: 0.83–0.98), patients who did not receive chemotherapy were 4% less likely (OR: 0.96, CI: 0.93–0.99), patients who did not receive RT were 4% less likely (OR: 0.96, CI: 0.93–0.99), and patients who did not receive immunotherapy were 10% less likely (OR: 0.90, CI: 0.83–0.97) to receive treatment at academic facilities compared to their counterparts. The characteristics of the patients and the OR of factors associated with receiving treatment at academic facilities are provided in Table 1.

**Table 1:** Multivariable logistic analysis with the probability of being treated in academic hospital in BMs patients

Variable		Academic 31,579 (34.1%)	Non-academic 61,054 (65.9%)	Total 92,633	OR (95% CI)	P
Age at diagnosis, Median (range)		64.00 (40–90)	66.00 (40–90)	65.00 (40–90)	0.99 (0.99–0.99)	0.001
Sex	Male	15,998 (50.7)	31,130 (51.0)	47,128 (50.9)	Reference	
	Female	15,581 (49.3)	29,924 (49.0)	45,505 (49.1)	1.02 (0.99–1.05)	0.23
Race	White	24,790 (79.4)	53,343 (87.9)	78,133 (85.0)	Reference	
	Black	5005 (16.0)	5596 (9.2)	10,601 (11.5)	1.94 (1.86–2.03)	0.001
	Other	1429 (4.6)	1785 (2.9)	3214 (3.5)	1.58 (1.47–1.70)	0.001
	Unknown	355	330	685		
Education	> = 13% NHD	15,228 (48.3)	28,622 (47.0)	43,850 (47.4)	1.07 (1.04–1.11)	0.001
	< 13% NHD	16,276 (51.7)	32,304 (53.0)	48,580 (52.6)	Reference	
	Unknown	75	128	203		
Income	> = $35,000	17,473 (55.5)	32,571 (53.5)	50,044 (54.2)	Reference	
	< 35,000	14,005 (44.5)	28,319 (46.5)	42,324 (45.8)	0.84 (0.81–0.87)	0.001
	Unknown	101	164	265		
Place of Living	Urban	30,510 (98.2)	57,724 (97.3)	88,234 (97.6)	Reference	
	Rural	547 (1.8)	1597 (2.7)	2144 (2.4)	0.73 (0.66–0.80)	0.001
	Unknown	522	1733	2255		
Insurance Status	Yes	29,057 (96.5)	56,998 (97.3)	86,055 (94.4)	Reference	
	No	1934 (3.5)	3195 (2.7)	5129 (5.6)	1.04 (0.98–1.11)	0.17
	Unknown	588	861	1449		
Charlson/Deyo Score	0	21,032 (66.6)	36,986 (60.6)	58,018 (62.6)	Reference	
	1	7132 (22.6)	16,130 (26.4)	23,262 (25.1)	0.82 (0.79–0.84)	0.001
	> = 2	3415 (10.8)	7938 (13.0)	11,353 (12.3)	0.81 (0.77–0.85)	0.001
Primary site surgery	Yes	1061 (3.4)	1604 (2.6)	2665 (2.9)	Ref	
	No	30,518 (95.6)	59,450 (97.4)	89,968 (97.1)	0.90 (0.83–0.98)	0.02
Chemotherapy	Yes	17,687 (56.0)	32,419 (53.1)	50,106 (54.1)	Reference	
	No	13,892 (44.0)	28,635 (46.9)	42,527 (45.9)	0.96 (0.93–0.99)	0.007
Radiation Therapy	Yes	23,373 (74.0)	43,737 (71.6)	67,110 (72.5)	Reference	
	No	8206 (26.0)	17,317 (28.4)	25,523 (27.5)	0.96 (0.93–0.99)	0.02
Immunotherapy	Yes	1217 (3.9)	1811 (3.0)	3028 (3.3)	Ref	
	No	30,362 (96.1)	59,243 (93.0)	89,605 (96.7)	0.90 (0.83–0.97)	0.009
Year of Diagnosis	2010–2013	20,224 (64.0)	40,100 (65.7)	60,324 (65.1)	0.93 (0.90–0.96)	0.001
	2014–2015	11,355 (36.0)	20,954 (34.3)	32,309 (34.9)	Reference	
Primary Cancer Type	Breast	14,141 (4.5)	2522 (4.1)	3936 (4.3)	0.69 (0.62–0.77)	0.001
	NSCLC	21,250 (67.3)	39,575 (64.8)	60,825 (65.7)	0.76 (0.70–0.83)	0.001
	SCLC	4406 (14.0)	10,378 (17.0)	14,784 (15.9)	0.64 (0.59–0.70)	0.001
	Other lung	1779 (5.6)	4450 (8.0)	6229 (6.7)	0.65 (0.59–0.72)	0.001
	Melanoma	1224 (3.9)	1825 (2.3)	3049 (3.3)	1.02 (0.92–1.15)	0.67
	Colorectal	393 (1.2)	726 (1.2)	1119 (1.2)	0.74 (0.64–0.86)	0.001
	Renal cell	1113 (3.5)	1578 (2.6)	2691 (2.9)	Ref	

*NHD* no high school degree

**Fig. 2:**
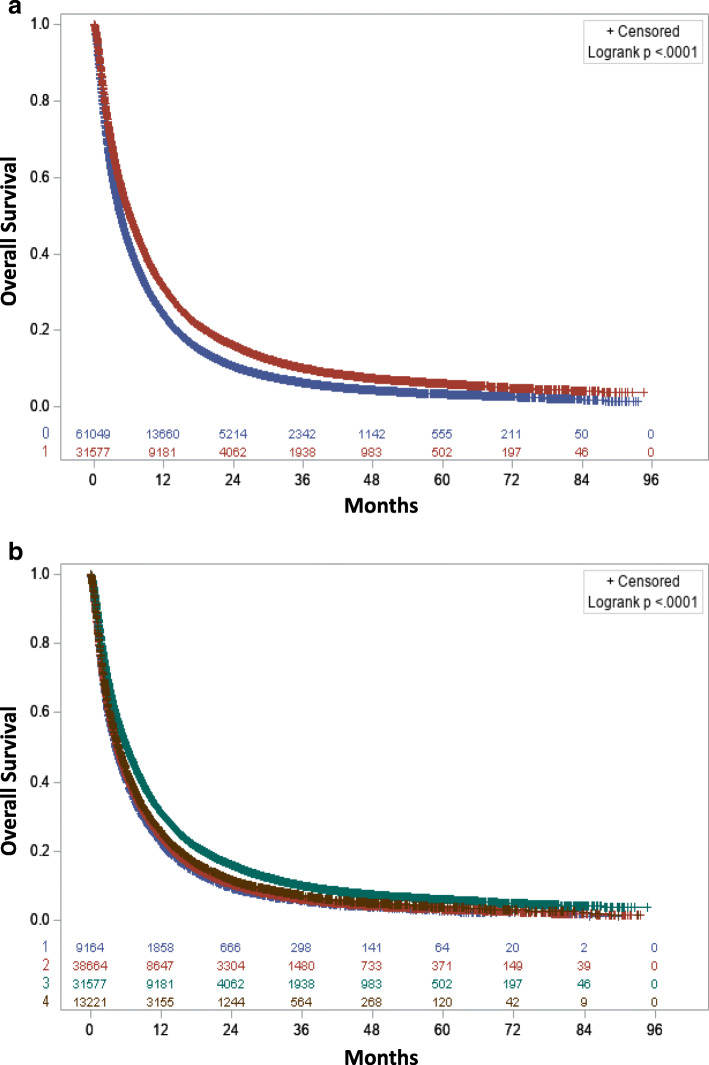
**a** Overall survival for patients receiving treatment at academic (AC-red) or non-academic centers (NAC-blue). **b** Overall survival for patients receiving treatment at Community Cancer Program (CCP-blue), Comprehensive Community Cancer Program (CCCP-red), Integrated Network Cancer Program (INCP-maroon), and academic Centers/Research (AC/R-green)

Based on the Kaplan Meier curves, patients who received treatment at an academic facility had significantly improved OS with an absolute median OS benefit of 1.61 (6.18, CI: 6.05–6.31 vs. 4.57, CI: 4.50–4.63; *p* < 0.001) months compared to patients who were treated at non-academic facilities (Fig. 2a). The 1-year and 2-year survival rates were 32% (CI: 31–32%) and 16% (CI: 16–17%) in patients treated at academic hospitals vs. 24% (CI: 24–25%) and 11% (CI: 10–11%) in patients treated at non-academic hospitals. The median OS of patients treated at academic hospitals was longer compared to patients who were treated at community hospitals among most of the treatment options. The KM curves by treatment options are provided in Supplemental Figures (1a-2d).

In the univariate Cox regression analysis, receiving treatment at academic hospitals, younger age, female sex, black race, non-white non-black race, living in areas with income = > $35,000, living in areas with high education level, living in the urban areas, having insurance, comorbidity score of 0, surgery of the primary cancer type, chemotherapy, RT, immunotherapy, diagnosis between 2014 and 2015 and primary cancer type of breast (renal cancer) were all associated with improved OS.

In the multivariable Cox regression analysis adjusted for age at diagnosis, race, sex, income level, education, place of living, insurance status, surgery of the primary site, chemotherapy, RT, immunotherapy, year of diagnosis, and primary cancer type, receiving treatment at an academic facility was associated with significantly improved OS (HR: 0.85, CI: 0.84–0.87; *p* < 0.001) compared to receiving treatment at a non-academic facility (Table 2). Other variables associated with significantly improved OS were young age, female sex, black race, non-white non-black race, having insurance, living in an area with an income level of = > $35,000, comorbidity score of zero, receiving surgery of the primary site, receiving chemotherapy, RT, immunotherapy, diagnosis in 2014 or after, and primary cancer type of breast, and melanoma (compared to kidney cell). The findings stayed significant after stratifying by comorbidity score and age of diagnosis. To make sure that the results of our study are not affected by immortal time bias, we conducted an analysis restricted to only patients who received all of the first-course treatment at the reporting facility. Treatment at an academic center still remained significantly associated with improved OS compared to treatment at a non-academic center (HR: 0.819, CI: 0.802–0.836; *p* < 0.001). The survival benefit of receiving treatment at an academic center became more significant compared to treatment at a non-academic center, an indication that our results underestimated the improved OS associated with receiving treatment at an academic center.

**Table 2:** Univariable and multivariable Cox proportional regression analysis of factors associated with OS in BMs patients

Variable		Univariable analysis		Multivariable analysis	
		Hazard Ratio (95% CI)	P	Hazard Ratio (95% CI)	P
Age at diagnosis (continuous)		1.02 (1.02–1.03)	0.001	1.01 (1.01–1.01)	0.001
Facility Type	Academic	0.81 (0.80–0.82)	0.001	0.85 (0.84–0.87)	0.001
	Non-academic	Ref			
Sex	Male	Ref		Ref	
	Female	0.82 (0.81–0.83)	0.001	0.86 (0.85–0.88)	0.001
Race	White	Ref		Ref	
	Black	0.95 (0.93–0.97)	0.001	0.94 (0.91–0.96)	0.001
	non-white non-black	0.69 (0.67–0.72)	0.001	0.73 (0.70–0.76)	0.001
Education	> = 13% NHD	1.07 (1.05–1.08)	0.001	…	
	< 13% NHD	Ref			
Income	> = $35,000	Ref		Ref	
	<$35,000	1.12 (1.10–1.13)	0.001	1.06 (1.04–1.07)	0.001
Place of Living	Urban	Ref		Ref	
	Rural	1.08 (1.04–1.134)	0.004	….	
Insurance Status	Insured	Ref			
	Not insured	1.04 (1.01–1.07)	0.02	1.11 (1.08–1.15)	0.001
Charlson/Deyo Score	0	Ref		Ref	
	1	1.25 (1.24–1.28)	0.001	1.14 (1.13–1.16)	0.001
	> = 2	1.50 (1.47–1.53)	0.001	1.23 (1.20–1.26)	0.001
Primary Site Surgery	Yes	Ref	0.001	Ref	0.001
	No	2.24 (2.14–2.34)		2.13 (2.03–2.23)	
Chemotherapy	Yes	Ref		Ref	
	No	2.37 (2.34–2.40)	0.001	2.19 (2.15–2.22)	0.001
Radiation Therapy	Yes	Reference		Reference	
	No	1.62 (1.59–1.64)	0.001	1.25 (1.22–1.27)	0.001
Immunotherapy	Yes	Ref		Ref	
	No	1.91 (1.83–1.99)	0.001	1.45 (1.39–1.52)	0.001
Year of Diagnosis	2010–2013	1.09 (1.07–1.10)	0.001	1.06 (1.05–1.08)	0.001
	2014–2015	Ref		Ref	
Primary Cancer Type	Breast cancer	0.72 (0.68–0.76)	0.001	0.74 (0.70–0.78)	0.001
	NSCLC	1.09 (1.04–1.13)	0.001	1.06 (1.02–1.11)	0.009
	SCLC	1.20 (1.15–1.25)	0.001	1.24 (1.19–1.30)	0.001
	Other lung	2.26 (2.16–2.38)	0.001	1.39 (1.32–1.46)	0.001
	Melanoma	1.01 (0.95–1.07)	0.77	0.78 (0.74–0.83)	0.001
	Colorectal cancer	1.20 (1.11–1.29)	0.001	1.26 (1.166–1.36)	0.001
	Renal cell	Ref		Ref	

*NHD* no high school degree Ref = reference

We also performed the stratified analysis by treatment and compared the OS of patients treated at academic hospitals vs. non-academic hospitals. Among patients who only received brain RT, receiving treatment at an academic facility was associated with significantly improved OS (HR: 0.84, CI: 0.82–0.87; *p* < 0.001) compared to receiving treatment at a non-academic facility. Treatment at academic hospitals was associated with improved OS compared to treatment at non-academic hospitals among patients who only received RT to other than brain (HR: 0.88, CI: 0.83–0.93; *p* < 0.001), patients who received chemotherapy plus brain RT (HR: 0.85, CI: 0.83–0.87; *p* < 0.001), and patients who received surgery of the primary site plus chemotherapy plus brain RT (HR: 0.75, CI: 0.64–0.88; *p* < 0.003). The HR of academic vs. non-academic stratified by various treatment combinations is provided in Table 3. We further compared survival outcomes for academic comprehensive cancer programs (ACCPs), integrated network cancer programs (INCPs), comprehensive community cancer programs (CCCPs), and community cancer programs (CCPs) to check if our findings stand beyond the academic vs. non-academic facilities. Patients treated at academic comprehensive cancer programs had significantly improved OS compared to each of the other types of facilities (Fig. 2b). The findings stayed significant when stratified by comorbidity score and age of diagnosis (data not shown). In terms of the technique of RT, when we stratified the RT method to WBRT and SRT, the results stayed the same. Treatment at the academic facility was associated with improved OS compared to treatment at non-academic centers among patients who received WBRT regardless of other treatments (HR: 0.882, CI: 0.858–0.907; *P* < 0.001) and among patients who received SRT irrespective of other treatments (HR: 0.921, CI: 0.873–0.972; *P* < 0.003). When we dichotomized the year of diagnosis to 2010–2012 (48%) and 2013–2015 (52%), the results of the treatment at academic centers vs. non-academic centers stayed the same (HR: 0.853, CI: 0.840–0.866; *P* < 0.001). Additionally, when we used the year of diagnosis as an individual year, the results stayed the same (HR: 0.853, CI: 0.840–0.866; *P* < 0.001). When we performed multivariable Cox regression analysis stratified by primary tumor type, the results stayed the same for all tumor types except colon cancer. The results are provided in Table 4. Also, when we performed a 1:1 (30,085 cases and 30,085 controls) Propensity score-matched analysis, receiving treatment at academic centers was associated with improved OS compared to treatment at non-academic centers (HR: 0.855, CI: 0.840–0.869; *P* < 0.001).” (data not shown).

**Table 3:** Multivariable Cox analysis of academic vs. non-academic (reference) stratified by treatment combinations

Combinations	N (%)	Multivariable Cox analysis HR ((95% CI)	
Only brain RT	17,879 (19.7)	0.84 (0.82–0.87)	0.001
Only other RT	5790 (6.4)	0.88 (0.83–0.93)	0.001
Only chemotherapy	7033 (7.8)	0.83 (0.78–0.88)	0.001
Only surgery	335 (0.4)	…	
Chemotherapy plus brain RT	31,341 (34.5)	0.85 (0.83–0.87)	0.001
Chemotherapy plus other RT	9663 (10.7)	0.86 (0.82–0.90)	0.001
Surgery plus brain RT	660 (0.7)	0.78 (0.64–0.94)	0.009
Surgery plus other RT	78 (0.1)	…	
Surgery plus chemotherapy	280 (0.3)	0.74 (0.55–0.99)	0.046
Surgery plus chemotherapy plus brain RT	1063 (1.2)	0.75 (0.64–0.88)	0.003
Surgery plus chemotherapy plus other RT	210 (0.2)	…	
No surgery no chemotherapy no RT	16,398 (18.0)	0.91 (0.88–0.95)	0.001

*RT* radiation therapy; The results of only surgery, surgery plus other RT, and surgery plus chemotherapy plus other RT were not significant

**Table 4:** Univariable and multivariable analyses of Academic vs. Non-academic stratified by primary cancer site

Tumor type	Univariable HR (95% CI)	P	Multivariable HR (95% CI)	P
	Academic centers vs. non-academic centers		Academic centers vs. non-academic centers	
Breast cancer	0.838 (0.778–0.902)	0.001	0.848 (0.785–0.916)	0.001
NSCL	0.812 (0.798–0.827)	0.001	0.854 (0.838–0.869)	0.001
SCLC	0.876 (0.844–0.909)	0.001	0.902 (0.868–0.937)	0.001
Other types of lung cancer	0.784 (0.740–0.830)	0.001	0.846 (0.796–0.898)	0.001
Melanoma	0.733 (0.678–0.794)	0.001	0.781 (0.719–0.847)	0.001
Colon cancer	0.907 (0.796–1.033)	0.14	0.945 (0.822–1.086)	0.42
Kidney cancer	0.761 (0.700–0.827)	0.001	0.803 (0.736–0.876)	0.001

Multivariable analyses were adjusted for age at diagnosis, sex, race, health insurance status, income level, education level, place of living, comorbidity score, chemotherapy, radiation therapy, immunotherapy, surgery to the primary cancer site, and year of diagnosis

## Discussion

This study is the most extensive retrospective study of the NCDB, analyzing the impact of treatment facility on the OS of patients diagnosed with BMs regardless of the primary cancer site. In the current study, BMs patients who were treated at an academic facility had better OS compared to patients who were treated at a non-academic facility. Another key finding of the current study was the association between the patient characteristics and social determinants of health, such as race, income, place of living, and where a patient would receive treatment. To our knowledge, there has not been any study that has investigated the impact of treatment at an academic hospital on the OS of BMs patients regardless of the primary cancer type. This study is important because by knowing the fact that receiving treatment at academic centers was associated with significantly improved OS compared to treatment at non-academic centers, we will be able to further identify and possibly remove negative factors associated with the poor survival outcome at non-academic center which will lead to an improved survival outcome at non-academic centers.

The results of the current study are consistent with the findings of other studies in various cancers [25–28]. A study of glioblastoma patients who received chemoradiation therapy after surgical resection or biopsy reported improved OS for patients treated at academic facility compared to patients treated at community hospitals (HR: 0.86, CI: 0.82–0.91) [27]. Receiving treatment at an academic hospital was associated with improved OS in a study of patients age ≥ 70 diagnosed with glioblastoma (HR: 0.76, CI: 0.66–0.86) compared to receiving treatment at non-academic facilities [26]. Further, a study of glioblastoma patients who received surgery reported worse OS (HR: 1.07, CI: 1.05 1.09, and 1.13, CI: 1.10 1.15) for Comprehensive Community Cancer programs and Integrated Network Cancer programs compared to academic centers [28]. Head and neck cancer patients with the surgical resection of the tumor who received treatment at academic hospitals had better OS compared to patients treated at community hospitals (HR: 0.89, CI: 0.88–0.91) [25].

Patients with BMs are different from primary brain tumors and patients of head and neck cancers. The pathophysiology, prevalence, and treatment approaches vary significantly. Treatment of primary cancer type and treatment of brain metastases both may play a critical role in the OS of BMs patients. The OS of these patients may be improved via the treatment or control of the primary cancer type or the control of the intracranial tumor. The improved OS associated with receiving treatment at academic hospitals in the current study could be due to several reasons. Improved OS perhaps reflects the expertise, availability of clinical trials, and multidisciplinary care for BMs patients at academic centers. Another important reason is the use of different treatment techniques in academic hospitals vs. non-academic hospitals. Academic centers are more likely to use RT to any site, chemotherapy, surgery, and brain RT. [34] Academic centers were more likely to use stereotactic radiosurgery vs. community facility (OR 1.76, CI 1.66–1.87, *p* < .001) for patients diagnosed with BMs from NSCLC [23]. In our study, academic centers were more likely to use chemotherapy (56% vs. 53%) and RT (74% vs. 71%) compared to non-academic centers. Chemotherapy and RT are associated with improved OS in BMs patients. The positive impact of chemotherapy in these patients is more likely due to its impact on primary cancer.

It is also possible that academic centers treat healthier patients compared to non-academic centers. Patients with more favorable prognoses may be more willing to travel to an academic center for care. Academic centers had a slightly higher proportion of patients with a zero Charlson comorbidity score (67% vs. 61%). However, the 6% absolute difference is unlikely to be clinically significant, and it is less likely that the improved OS associated with receiving treatment at academic centers is biased due to the difference in the proportion of patients with different comorbidity scores. Patients treated at academic hospitals still had significantly improved OS compared to patients treated at non-academic hospitals when the analysis was stratified by comorbidity score. Differences in the patient and socioeconomic factors of BMs patients treated at academic vs. non-academic centers may be another potential explanation for the improved OS of patients treated at academic centers. However, this is less likely because the proportions of patients with insurance and belonging to high income and high education level areas were similar between academic and non-academic centers.

Access to care may be another explanation for the improved OS. In our study, patients from high-income levels and living in the urban areas were more likely to receive treatment at academic centers. Moreover, BMs patients who received chemotherapy, RT to any site, brain RT, surgery of the primary cancer site, and immunotherapy were more likely to be treated at academic centers compared to their counterparts who did not receive these treatments. All these treatments were associated with improved OS in the multivariable analysis indicating the importance of these treatments in the prognosis of BMs. Targeted therapies and immunotherapies have been associated with improved OS and intracranial response in BMs patients from various cancers. Patients who receive immunotherapy were also more likely to be treated at academic centers compared to patients who did not receive immunotherapy. However, only 3% of the patients in the current study received immunotherapy. More importantly, treatment at academic centers was associated with improved OS compared to treatment at non-academic centers when the analysis was stratified by immunotherapy. Targeted therapy is listed in the immunotherapy group in the NCDB.

In the current study, patients of the Black race and patients who were living in the areas with low education levels were more likely to be treated at academic centers. These findings are likely due to proximity bias (i.e., These patients were living in the areas where academic treatment facilities tend to be located). Black patients also had better OS compared to White, indicating if access to care is improved and if quality care is provided, Black patients can have comparable or even better survival than White patients. Studies of cancer patients in the Veterans Affairs health system with equal-access health systems have reported equal or even better OS in Black patients compared to White in patients with prostate cancer or multiple myeloma [35, 36]. The strength of the current study is its large sample size. With a large sample size, we were able to adjust for important factors that may confound the study findings.

### Limitations

Our study has several limitations. Patients may have visited more than one facility, and therefore it is difficult to delineate the real impact of facility type. The NCDB also not captures information about referral patterns and how patients select their treatment center. The study cannot provide information about which factor exactly contributes to advantageous survival outcomes. Is the improved OS due to RT, chemotherapy, chemoradiation, surgery, brain RT or combinations of many factors? The retrospective nature of the study and lack of information about cause-specific survival are also some limitations. Additional limitations include lack of information about brain surgery, number of tumors in the brain, size of the brain tumor, types of brain RT such as SRS, SRT, and WBRT, and whether RT was delivered only to the brain, other sites, or both. NCDB also does not have the information on the number of lines of treatment. Finally, the presence of unknown confounding factors that we were not able to adjust for in the database is an additional limitation of the study. Despite these limitations, the current study is the most extensive and includes the majority of the BMs patients treated in the United States. The NCDB is a powerful tool for reporting the survival comparisons between different groups of patients based on various treatment, patients, and tumor-related factors.

## Conclusions

In this robust and comprehensive retrospective analysis of the NCDB, BMs patients who received treatment at academic centers had significantly improved OS compared to patients who received treatment at non-academic centers. Certain socioeconomic factors and health disparities affect where a patient may get treatment. Future research should focus on determining the extent to how much the different patient populations, socioeconomic factors, and provider expertise contribute to the disparities in the OS of patients treated at academic centers compared to non-academic centers.

## Supplementary Information

**Additional file 1: Supplemental Figure 1.** Overall survival with receiving treatment at academic centers (red) or non-academic center (blue) for (A) patients who did not receive surgery or chemotherapy or RT, (B) patients who only received radiation therapy to the brain, (C) patients who only received radiation therapy to sites other than the brain, (D) patients who only received chemotherapy, and (E) patients who only received surgery of the primary cancer site.

**Additional file 2: Supplemental Figure 2.** Overall survival with receiving treatment at academic centers (red) or non-academic center (blue) for (A) patients who received chemotherapy plus radiation therapy to the brain, (B) patients who received chemotherapy plus radiation therapy to sites other than the brain, (C) patients who received surgery of the primary cancer type plus radiation therapy to the brain, and (D) patients who received chemotherapy plus surgery of the primary cancer type plus radiation therapy to the brain.

## Data Availability

The datasets used and analyzed during the current study are available from the corresponding author on reasonable request.

## Abbreviations

NCDB: National Cancer Database
SEER: Surveillance, Epidemiology, and End Results Program
BMs: Brain metastases
OS: Overall survival
RT: Radiation therapy
WBRT: Whole-brain radiation therapy
SRS: Stereotactic radiosurgery
NSCLC: Non-small cell lung cancer
SCLC: Small-cell lung cancer
CRC: Colorectal cancer
