# Supplementary figures and images for: The impact of treatment facility type on the survival of brain metastases patients regardless of the primary cancer type
Source: BMC Cancer. 2021 Apr 9;21:387. doi: 10.1186/s12885-021-08129-4 (PMC8033704; doi:10.1186/s12885-021-08129-4)

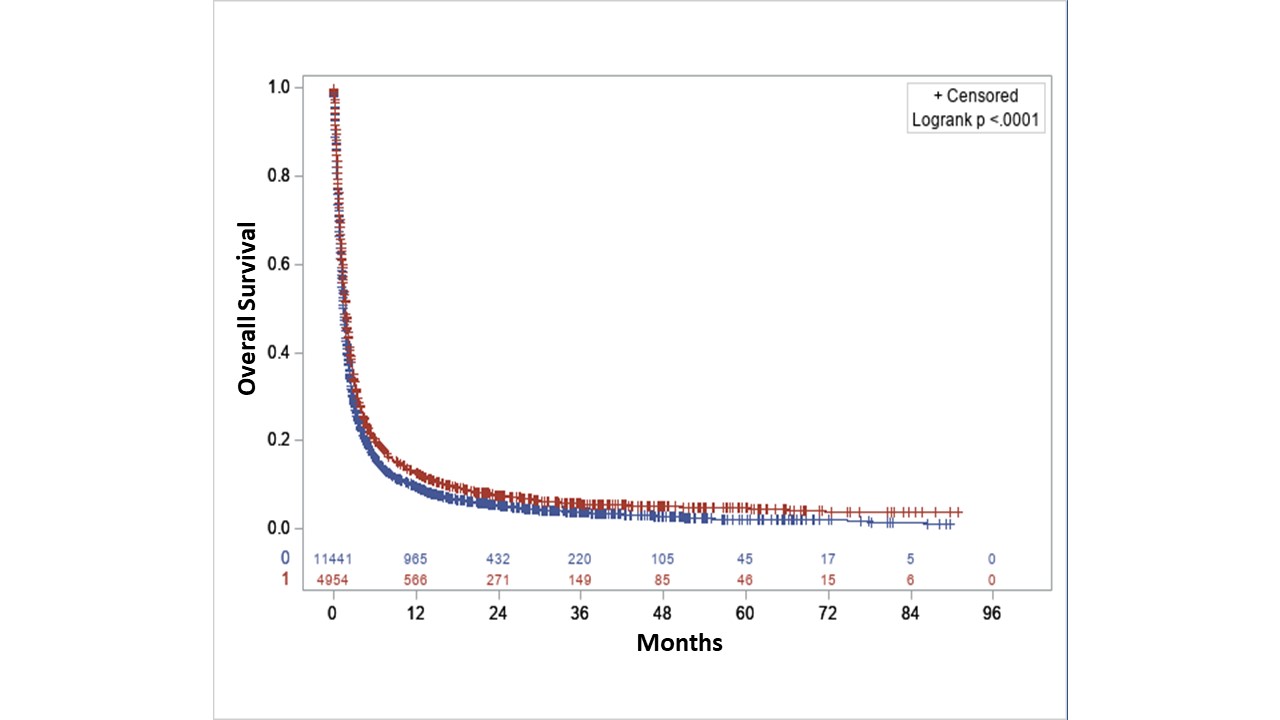

Supplement: Supplementary file 1 — Additional file 1: Supplemental Figure 1. Overall survival with receiving treatment at academic centers (red) or non-academic center (blue) for (A) patients who did not receive surgery or chemotherapy or RT, (B) patients who only received radiation therapy to the brain, (C) patients who only received radiation therapy to sites other than the brain, (D) patients who only received chemotherapy, and (E) patients who only received surgery of the primary cancer site. [file 12885_2021_8129_MOESM1_ESM.zip › Supplemental Figure 1a.jpg]

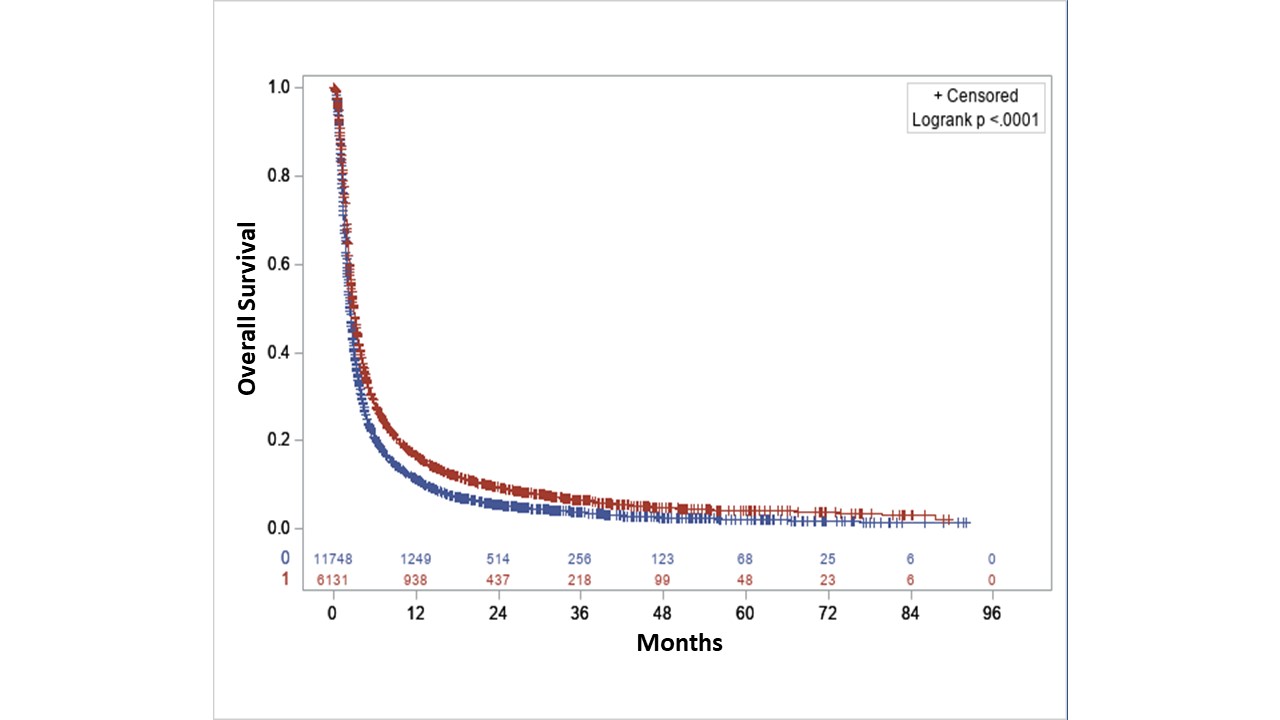

Supplement: Supplementary file 1 — Additional file 1: Supplemental Figure 1. Overall survival with receiving treatment at academic centers (red) or non-academic center (blue) for (A) patients who did not receive surgery or chemotherapy or RT, (B) patients who only received radiation therapy to the brain, (C) patients who only received radiation therapy to sites other than the brain, (D) patients who only received chemotherapy, and (E) patients who only received surgery of the primary cancer site. [file 12885_2021_8129_MOESM1_ESM.zip › Supplemental Figure 1b.jpg]

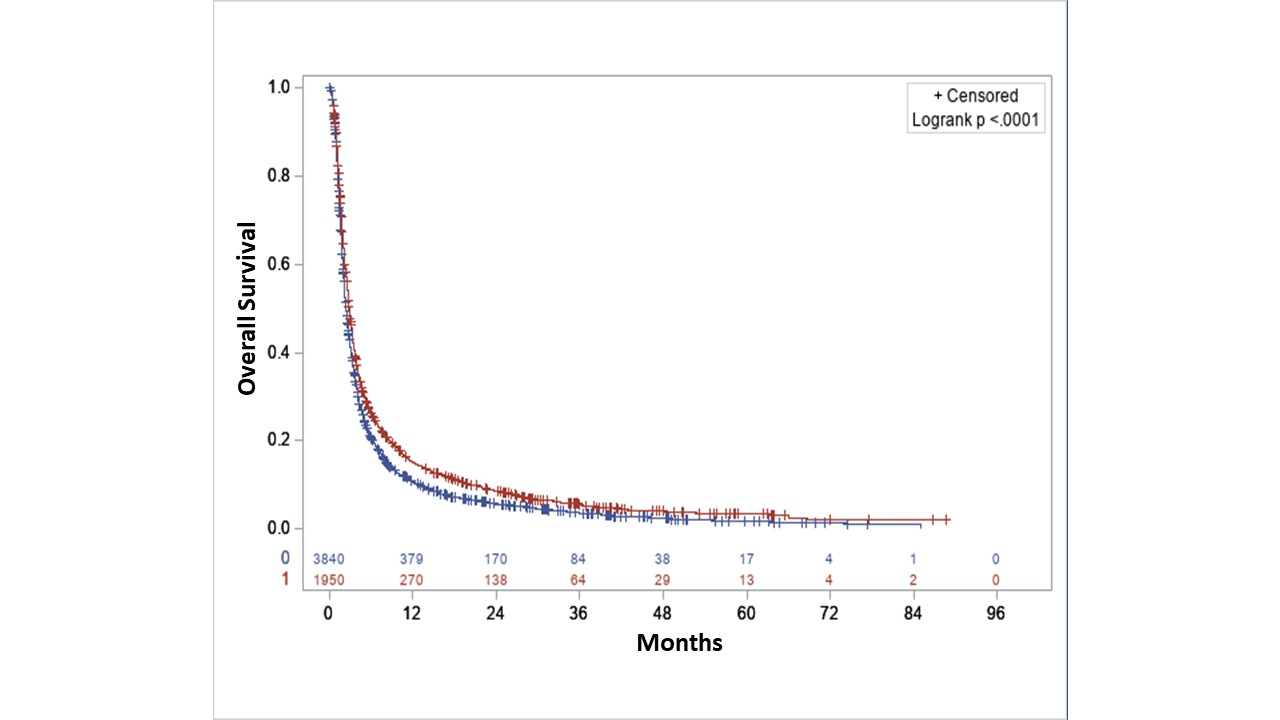

Supplement: Supplementary file 1 — Additional file 1: Supplemental Figure 1. Overall survival with receiving treatment at academic centers (red) or non-academic center (blue) for (A) patients who did not receive surgery or chemotherapy or RT, (B) patients who only received radiation therapy to the brain, (C) patients who only received radiation therapy to sites other than the brain, (D) patients who only received chemotherapy, and (E) patients who only received surgery of the primary cancer site. [file 12885_2021_8129_MOESM1_ESM.zip › Supplemental Figure 1c.jpg]

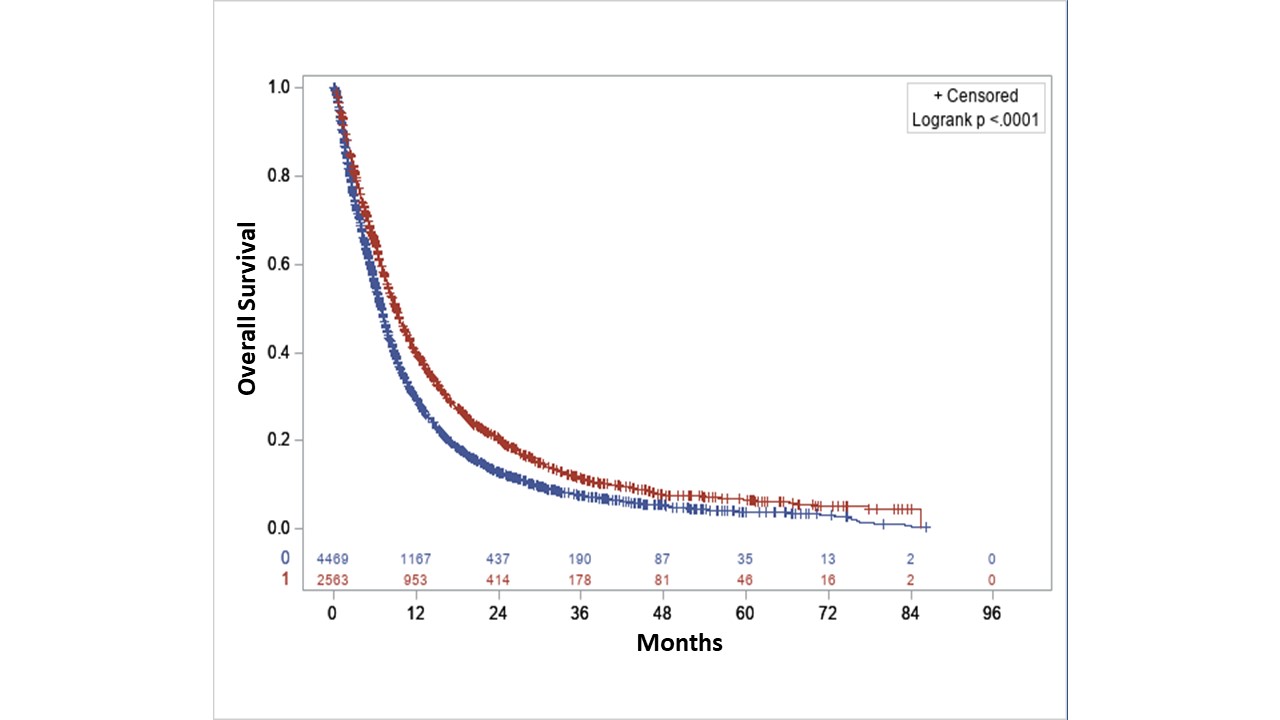

Supplement: Supplementary file 1 — Additional file 1: Supplemental Figure 1. Overall survival with receiving treatment at academic centers (red) or non-academic center (blue) for (A) patients who did not receive surgery or chemotherapy or RT, (B) patients who only received radiation therapy to the brain, (C) patients who only received radiation therapy to sites other than the brain, (D) patients who only received chemotherapy, and (E) patients who only received surgery of the primary cancer site. [file 12885_2021_8129_MOESM1_ESM.zip › Supplemental Figure 1d.jpg]

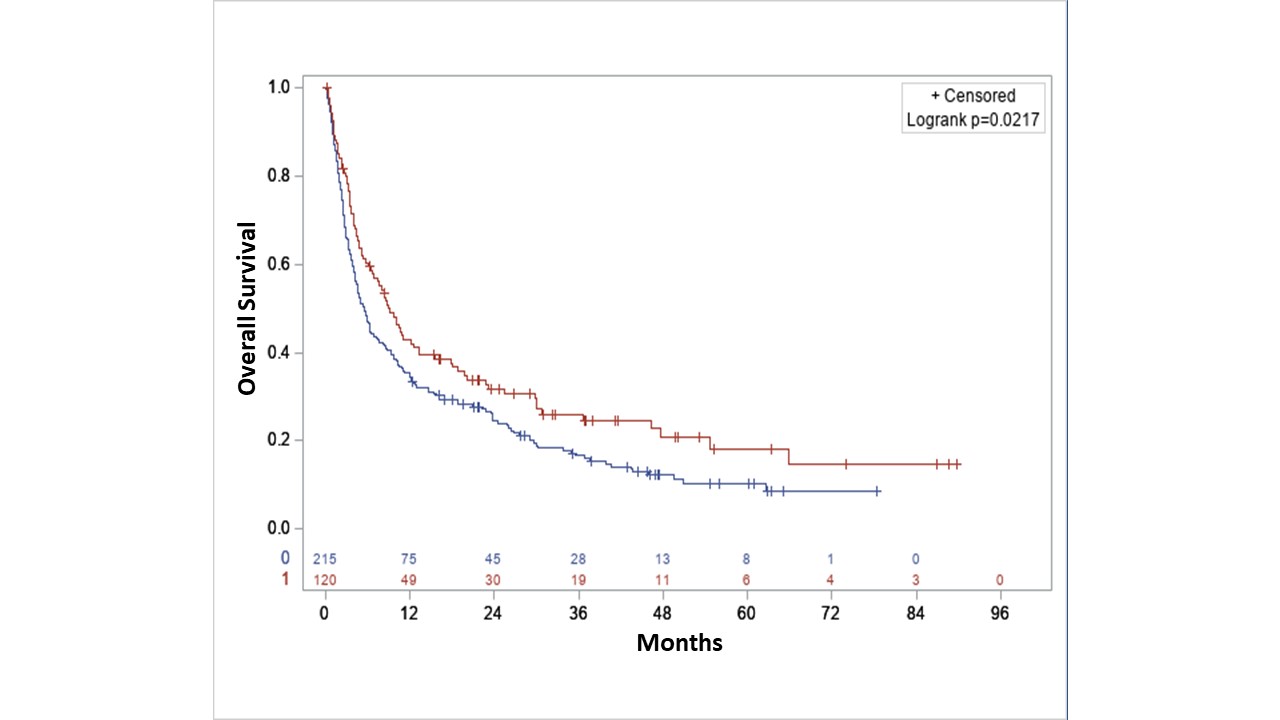

Supplement: Supplementary file 1 — Additional file 1: Supplemental Figure 1. Overall survival with receiving treatment at academic centers (red) or non-academic center (blue) for (A) patients who did not receive surgery or chemotherapy or RT, (B) patients who only received radiation therapy to the brain, (C) patients who only received radiation therapy to sites other than the brain, (D) patients who only received chemotherapy, and (E) patients who only received surgery of the primary cancer site. [file 12885_2021_8129_MOESM1_ESM.zip › Supplemental Figure 1e.jpg]

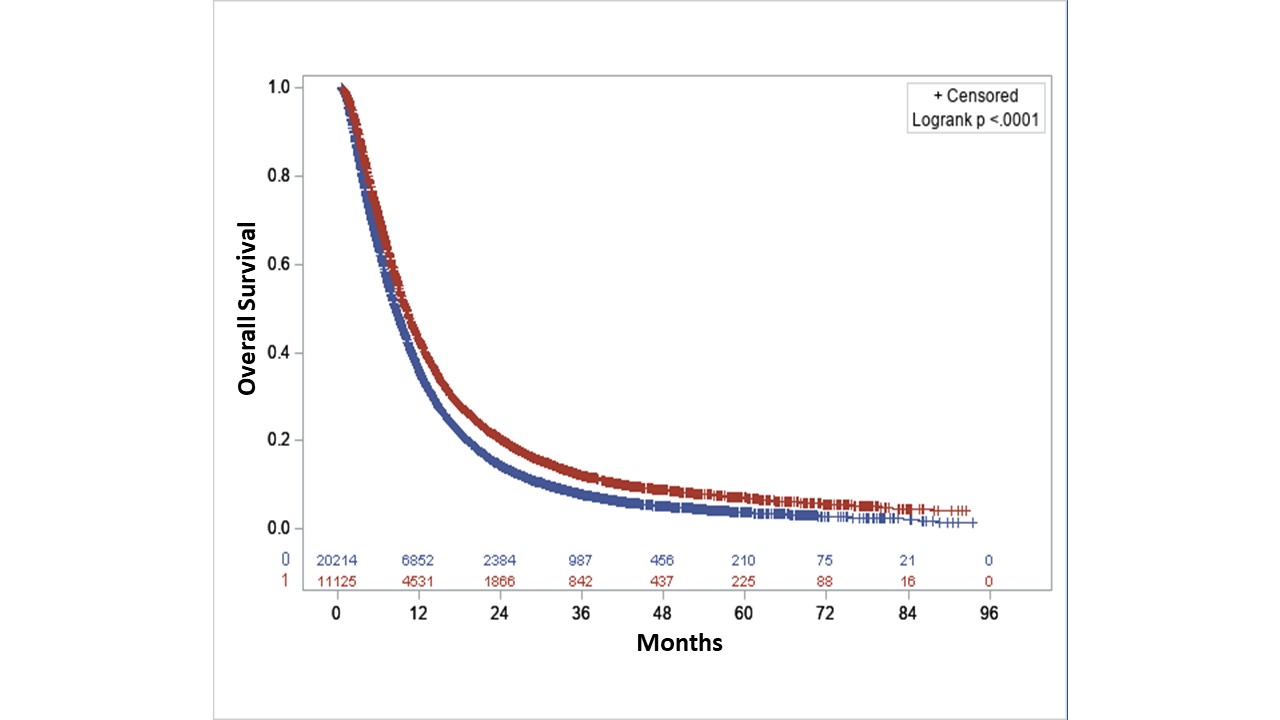

Supplement: Supplementary file 2 — Additional file 2: Supplemental Figure 2. Overall survival with receiving treatment at academic centers (red) or non-academic center (blue) for (A) patients who received chemotherapy plus radiation therapy to the brain, (B) patients who received chemotherapy plus radiation therapy to sites other than the brain, (C) patients who received surgery of the primary cancer type plus radiation therapy to the brain, and (D) patients who received chemotherapy plus surgery of the primary cancer type plus radiation therapy to the brain. [file 12885_2021_8129_MOESM2_ESM.zip › Supplemental Figure 2a.jpg]

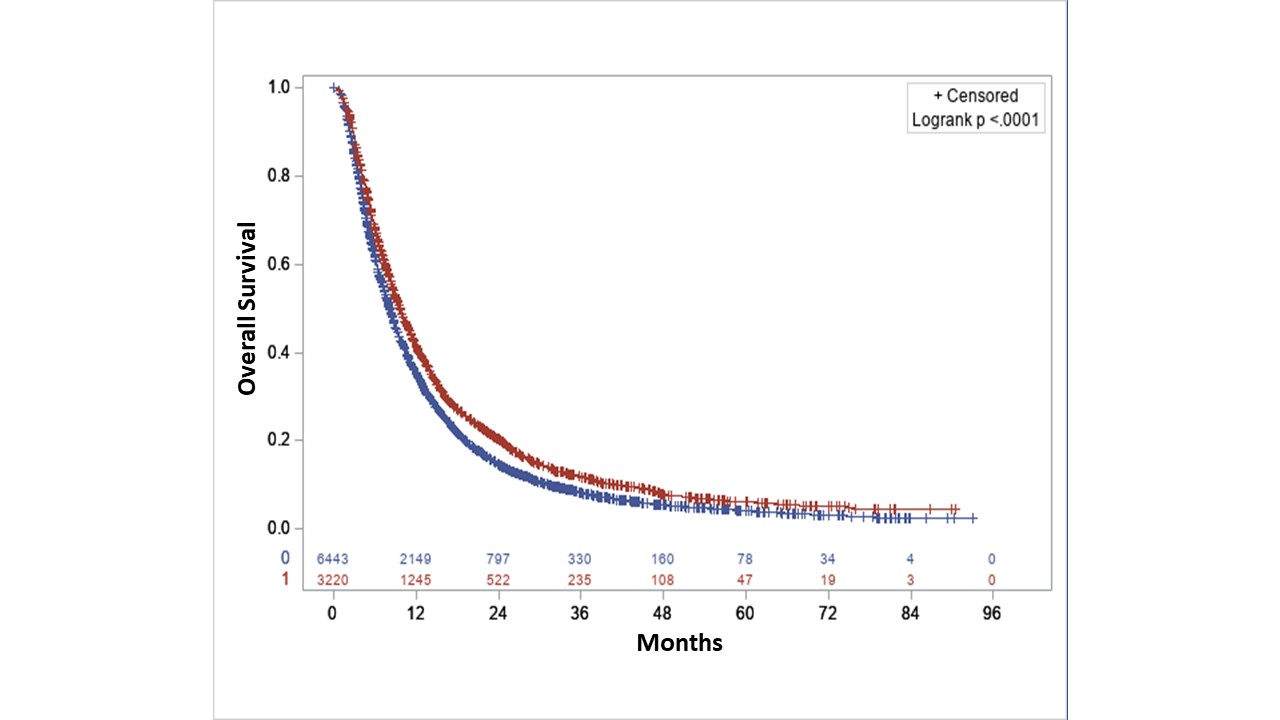

Supplement: Supplementary file 2 — Additional file 2: Supplemental Figure 2. Overall survival with receiving treatment at academic centers (red) or non-academic center (blue) for (A) patients who received chemotherapy plus radiation therapy to the brain, (B) patients who received chemotherapy plus radiation therapy to sites other than the brain, (C) patients who received surgery of the primary cancer type plus radiation therapy to the brain, and (D) patients who received chemotherapy plus surgery of the primary cancer type plus radiation therapy to the brain. [file 12885_2021_8129_MOESM2_ESM.zip › Supplemental Figure 2b.jpg]

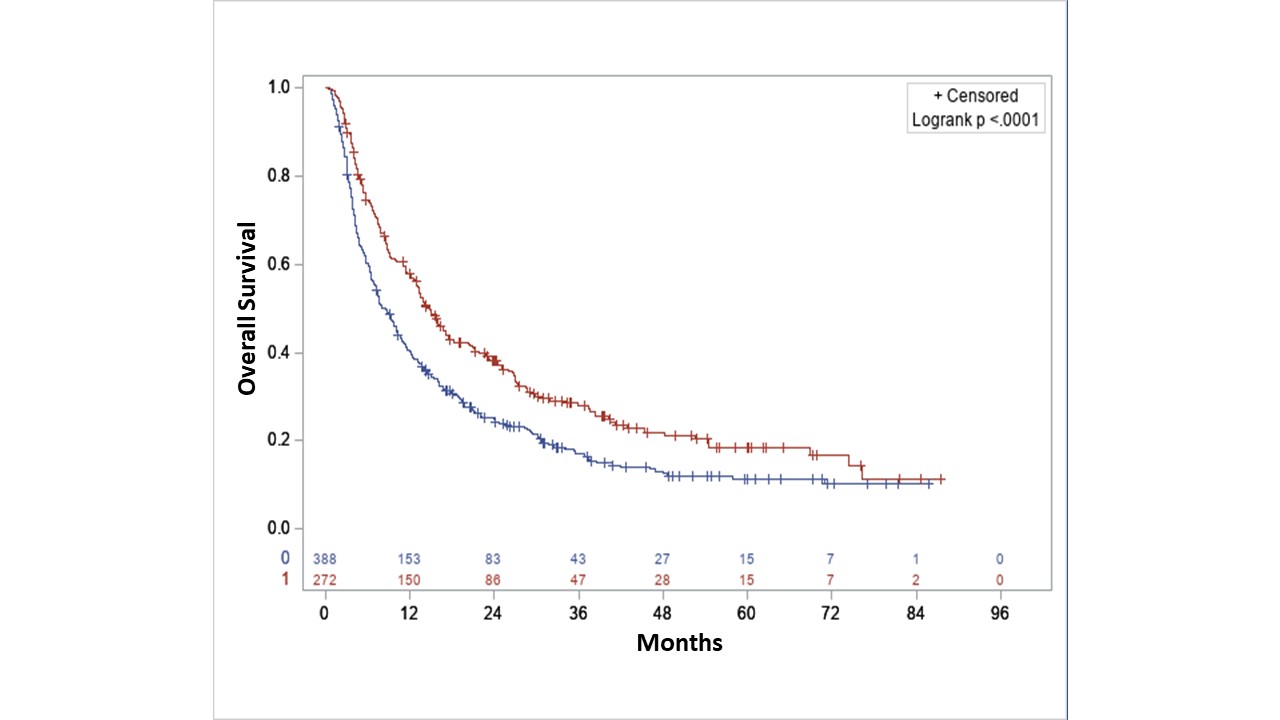

Supplement: Supplementary file 2 — Additional file 2: Supplemental Figure 2. Overall survival with receiving treatment at academic centers (red) or non-academic center (blue) for (A) patients who received chemotherapy plus radiation therapy to the brain, (B) patients who received chemotherapy plus radiation therapy to sites other than the brain, (C) patients who received surgery of the primary cancer type plus radiation therapy to the brain, and (D) patients who received chemotherapy plus surgery of the primary cancer type plus radiation therapy to the brain. [file 12885_2021_8129_MOESM2_ESM.zip › Supplemental Figure 2c.jpg]

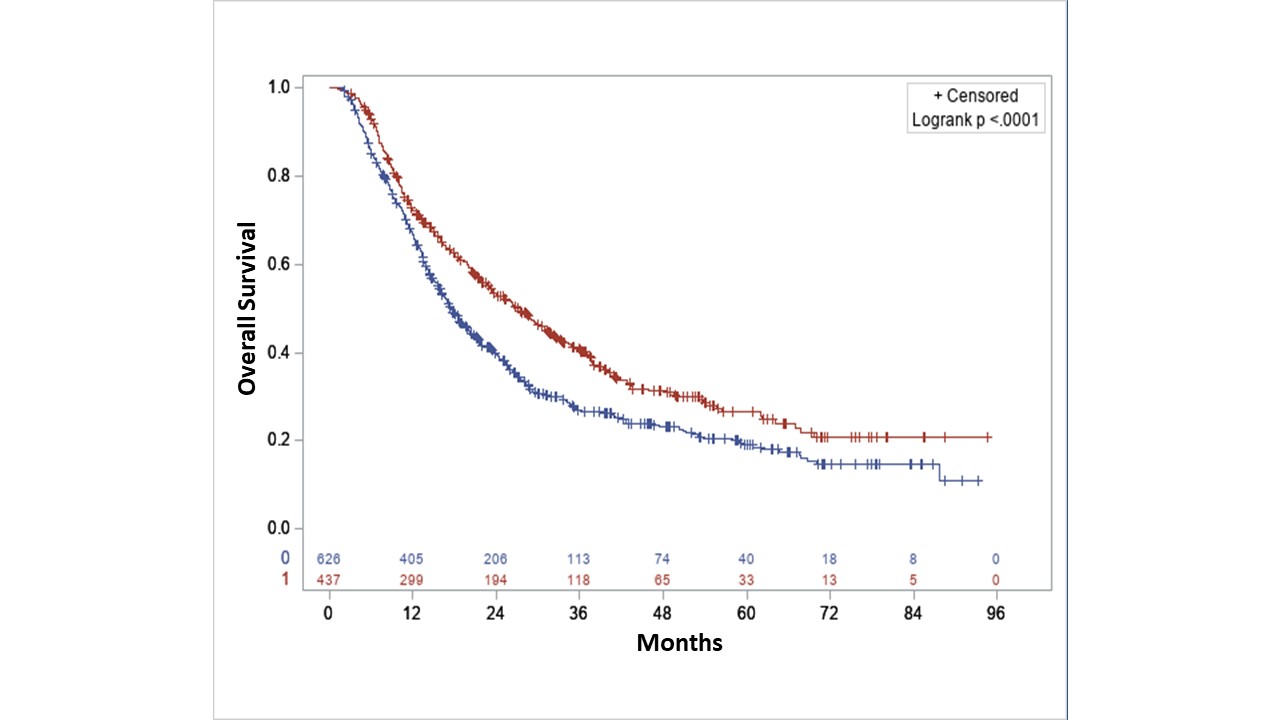

Supplement: Supplementary file 2 — Additional file 2: Supplemental Figure 2. Overall survival with receiving treatment at academic centers (red) or non-academic center (blue) for (A) patients who received chemotherapy plus radiation therapy to the brain, (B) patients who received chemotherapy plus radiation therapy to sites other than the brain, (C) patients who received surgery of the primary cancer type plus radiation therapy to the brain, and (D) patients who received chemotherapy plus surgery of the primary cancer type plus radiation therapy to the brain. [file 12885_2021_8129_MOESM2_ESM.zip › Supplemental Figure 2d.jpg]
